# Beta oscillations in major depression – signalling a new cortical circuit for central executive function

**DOI:** 10.1038/s41598-017-18306-w

**Published:** 2017-12-21

**Authors:** Yuezhi Li, Cheng Kang, Zhaoguo Wei, Xingda Qu, Tiebang Liu, Yunfei Zhou, Yong Hu

**Affiliations:** 1 Laboratory of Neural Engineering, Shenzhen University, Shenzhen, China; 2 Department of Orthopaedics and Traumatology, The University of Hong Kong, Pokfulam, Hong Kong; 3 Shenzhen Kangning Hospital, Shenzhen, China

## Abstract

This study aimed to examine alterations in electroencephalography (EEG) phase synchronization in working memory processing in depressed patients. Sixty-four-channel EEG signals were recorded from 33 depressed patients and 32 healthy controls during a visual n-back task. Alterations in functional connections in the patients were investigated using event-related phase coherence in terms of the phase synchronization index (PSI). Compared with the control subjects, the depressed patients showed a lower task-dependent increase in the PSI of delta, theta, and alpha oscillations in a frontoparietal network, but a higher task-dependent increase in the PSI of beta oscillations in the frontoparietal network. Additionally, depressed patients showed a lower task-dependent decrease in the PSI of delta, theta, alpha, and beta oscillations in centro-parieto-occipital sites. Insufficient phase synchronization and desynchronization during working memory processing reflects impairments in cortical inhibition, memory, and attention efficiency in major depression, while the abnormal increase in phase synchronization in beta oscillations in the frontoparietal network may indicate a new cortical circuit concerned with the repair of impaired ability in attention, memory retention, and working memory central executive processing. These findings present a compensatory mechanism for impaired cognitive function in major depression, and advance our understanding of functional aspect of beta oscillations.

## Introduction

Major depression is characterized by dysfunctions in attention and difficulties in concentration and decision making. Depressed patients show an inability to inhibit neutral information access to working memory and restrain irrelevant information processing; leading to cognitive slowness and attentional deficits. This profile of major depression may be attributable to a deficit in the efficacy of the central executive component of human working memory^1,2^. An fMRI investigation into working memory in major depression found that depressed patients demonstrated greater activation of the lateral prefrontal cortex and the anterior cingulate while maintaining a similar level of performance to controls during a working memory task^3^. Another fMRI study observed greater activation of the medial orbitofrontal and rostral anterior cingulate in maintenance of ‘normal’ working memory performance in major depression^4^. However, the contribution of these greater activations to performance of working memory is not well understood. Within the above context, a network-based study to investigate deficits and compensatory activation in the working memory processing network is therefore important and necessary, and would advance our understanding of working memory processing in major depression.

EEG oscillations are rhythmic electrical events in the brain that emerge from the interactions of large populations of neurons^5,6^. Oscillatory activity has been suggested as constituting a mechanism for regulating the states of neuronal networks and specifying the mode of information processing^7^. The brain determines communication through neuronal pools at any particular moment by changing the frequency content of oscillations, and oscillations act as communication networks through large populations of neurons^8–10^. The phases of oscillations are directly related to the timing of neural activity, and the consideration of phase synchronization offers a basis for the understanding of the interrelations between different brain structures, which in turn emphasizes the functional-topographic aspect of different neuronal units^11^.

Major depression is suggested to affect activity in the thalamocortical and corticocortical circuits, which is reflected in altered EEG oscillations^7^. In the resting-state condition, major depression is characterized by unique EEG oscillations in beta frequencies that are dominant in relation to delta, theta, and alpha oscillations^7^, and this oscillatory pattern was never seen in healthy subjects. Another study has reported increased theta and beta phase synchronization in major depression related to attentional deficit in the visual oddball task^12^. In the present study, we hypothesized that disorganization of oscillation synchronization with abnormal theta and beta oscillation synchronization would be identifiable in a neuronal network involved in working memory processing in major depression, and that this would provide new insights into the unstable state of cognitive processing and advance our understanding of functional aspect of beta oscillations in the brain.

To identify the neuronal network involved in working memory processing in patients with major depression, we measured 64-channel EEG signals during a visual n-back task that utilized 0-back and 2-back memory load conditions. The event-related phase coherence (ERPCOH), as defined by the phase synchronization index (PSI)^13,14^, was calculated for the network. This study analyzed the PSI of a broad EEG frequency band (1–30 Hz) for 64 electrode sites. Firstly, the PSI value of each electrode pair was computed for the 2-back and 0-back conditions to define significant pairs. Secondly, the PSI values of significant pairs in patients with major depression and healthy controls were compared within the frequency ranges of 1 to 30 Hz at intervals of 1 Hz. Thirdly, a neuronal network was established on the basis of the PSI values, to identify the alterations in phase synchronization connections in major depression.

## Methods

### Participants

Thirty-two healthy subjects were recruited from the hospital or university staff, while 33 patients with major depression were recruited from the Depression and Anxiety Disorders Clinic at Shenzhen Kangning Hospital. All subjects were screened by two senior psychiatrists with more than 10-years experience, using Structured Clinical Interview for DSM-IV Axis I Disorders, Clinician Version (SCID-CV)^15^, the 17-item Hamilton Depression Rating Scale, and the Hamilton Anxiety Rating Scale. In the control group, all subjects passed the SCID-CV examination without history of depression. In the patient group, subjects with anxiety disorder, or a history of head trauma were excluded. Seventeen of the 33 patients were naive to antidepressants and no patient had received antidepressant treatment within the 4 weeks before the study. The patients were ever treated with either paroxetine (20–40 mg/day), venlafaxine (75–225 mg/day), or citalopram (20–40 mg/day). Table 1 shows the characteristics of the subjects. This study was performed in accordance with relevant guidelines and regulations approved by the institutional review board of Shenzhen University and written informed consent was obtained from each subject before the experiment.

**Table 1:** Demographic and clinical characteristics and n-back task performance of depressed patients and control subjects.

	Depressed patients (n = 33)	Control subjects (n = 32)
Age (years)	32.8 (8.9)	29.5 (6.2)
Education (years)	15.9 (2.6)	16.8 (1.8)
Sex ratio	14 F/9 M	11 F/11 M
HDRS-17	23.0 (4.3)	2.3 (1.7)
HARS	11.8 (4.9)	4.6 (1.5)
0-back		
Accuracy (%)	97.5 (3.3)	98.6 (1.4)
Reaction times (ms)	529.1 (87.8)	544.5 (73.4)
2-back		
Accuracy (%)	78.2 (14.6)	83.3 (9.7)
Reaction times (ms)	833.5 (184.6)	809.8 (176.1)

The severity of depression and anxiety was quantified by using HDRS-17 and HARS. HDRS-17 = Hamilton Depression Rating Scale (17 items); HARS = Hamilton Anxiety Rating Scale. There were no significant differences in age (t_63_ = 1.730; P = 0.095) or education level (t_63_ = 1.618; P = 0.116) across groups, and all groups had equivalent gender distribution.

### The n-back Task

A letter variant version of the n-back task was used in this experiment. All subjects performed 2-back and 0-back versions of the n-back task, which involved the subjects observing stimuli on a 23 inch computer monitor and responding by pressing a button with the index finger for a match (target stimulus), and with the middle finger for a mismatch (nontarget stimulus). In the 0-back condition, subjects were asked to identify the single pre-specified letter ‘X’, while in the 2-back condition, subjects were asked to identify whether a letter presented on a screen matched the letter presented 2 trials back. Presented letters were randomly selected from English consonants. All blocks consisted of a pseudo-random sequence of 30 consonants (10 target and 20 nontarget stimuli). Letters were presented for 0.5 s and were separated from one another by a 2-s delay. The total duration of a given block was 75 s, with each condition consisting of 8 blocks. Blocks were separated from one another by a 45-s interval. The different condition blocks were also presented pseudo-randomly. The task was programmed using E-prime software (Psychology Software Tools, Pittsburgh, PA). The subjects were instructed to respond as quickly and accurately as possible, and their responses (reaction time and response accuracy) were recorded, with only those trials with correct responses being included in the EEG analysis. The subjects were allowed to practice the tasks before the experiment using a practice block containing 30 trials; this could be repeated until the subjects were convinced that each condition in the task was clear. The response accuracy and reaction times were compared between the depression and control groups using Student’s *t*-tests.

### EEG Recording

Sixty-four-channel EEG signals were recorded using a BrainAmp amplifier (Brain Products, Munich, Germany). The electrodes were placed according to the 10–20 System and intermediate sites. An additional electrode, Iz, was placed on the infraorbital ridge of the right eye to record the vertical electrooculogram. All channels were referenced during recording to electrode FCz with a forehead ground (AFz). Electrode impedance was maintained below 5k Ω throughout the experiment. The EEG and electrooculogram were recorded without filtering, and digitized at a sampling rate of 1000 Hz.

### EEG Data Analysis

The EEG was digitally filtered with a band-pass filter of 0.16–50 Hz (24 dB/Octave). Electrooculogram artifacts were corrected by ocular correction using the independent components analysis algorithm in Brain Vision Analyzer software (Brain Products). EEG data referenced to FCz were recalculated against the average reference, and a time epoch for each event of 2500 ms (200 ms pre-stimulus and 2300 ms post-stimulus) was used. For each epoch, a baseline correction was performed according to the data 200 ms prior to the stimulus. To avoid eye movement and other artifacts, all epochs exceeding ±120 μV in any channel were excluded from additional analysis.

To remove the effects of volume conduction and reference signals on the phase synchronization measurements^13,14^, EEG signals were transformed to the scalp current density at each electrode site by applying a spherical Laplace operator to the voltage distribution on the scalp using Brain Vision Analyzer software. This transformation was performed using the following settings, order of the splines = 4, maximum degree of the Legendre polynomial = 10, precision = 10^−5,^ ^16^. After the signals of scalp current density at each electrode site were down-sampled to 500 Hz and exported to EEGLAB, The following further analysis was performed within the MATLAB environment (Mathworks, Natick, MA, USA).

The signal of the scalp current density was convoluted by a complex Morlet’s wavelet w(*f*, *t*):

1 $${\rm{w}}(f,t)=(\frac{1}{\sqrt{\pi {\delta }_{t}}}\exp (-{t}^{2}/2{\delta }_{t}^{2})\exp (j2\pi ft)$$

2 $${F}_{k}^{n}(f,t)={S}_{k}^{n}(t)\otimes w(f,t)$$

where w(*f*, *t*) is the product of a sinusoidal wave at frequency *f*, with a Gaussian function with a standard deviation *δ*_*t*_^17^. For computation in EEGLAB, the number of wavelet cycles was set to 0.5, and the lowest frequency time window to 0.5 s; this resulted in a lowest analysis frequency of 1 Hz, with the number of wavelet cycles used for higher frequencies continuing to expand using the fixed time window of 0.5 s. ${S}_{k}^{n}$ is the scalp current density of trial *k* at electrode *n*; ${F}_{k}^{n}(f,t)$ is the time-frequency transform of ${S}_{k}^{n}$ at frequency *f* and time *t*.

The PSI was then defined between electrode *l* and *m* with the following equation

3 $$PS{I}_{l,m}(f,t)=\frac{1}{n}\sum _{k=1}^{n}\frac{{F}_{k}^{l}(f,t){F}_{k}^{m}{(f,t)}^{\ast }}{|{F}_{k}^{l}(f,t){F}_{k}^{m}(f,t)|}$$

where *n* is the number of available trials. *PSI*_*l*,*m*_(*f*, *t*) is computed in 1 Hz steps from 1 Hz to 30 Hz. The set of *PSI*_*l*,*m*_(*f*, *t*) is termed PSI below.

**Figure 1:**
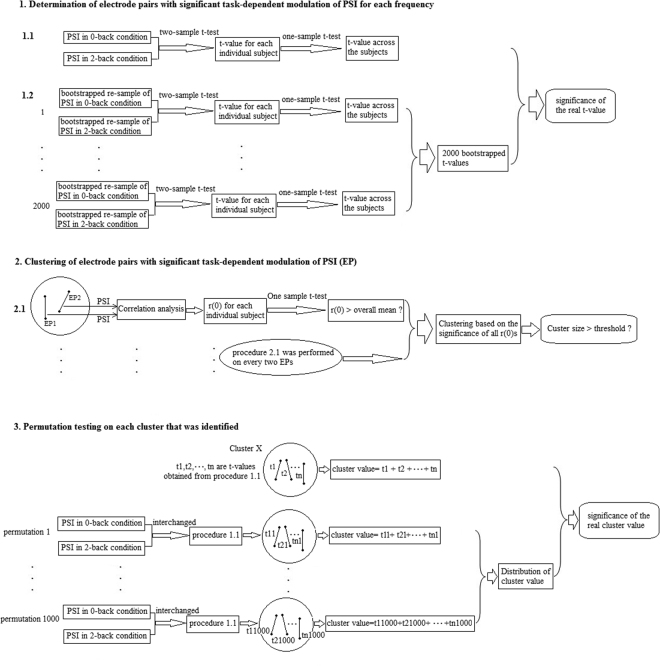
Processing flow for identification of significant electrode pairs, clustering and cluster-based statistical analysis.

To identify the task-dependent modulation of the PSIs, the time series samples of *PSI*_*l*,*m*_(*f*, *t* = [0 ms, 2300 ms]) from each individual subject were compared between the 2-back and 0-back conditions using two-sample *t*-tests. A one-sample *t*-test was performed on the acquired *t*-values from the two-sample *t*-tests to determine the task-dependent modulation of the PSIs across the subjects. A bootstrap procedure was then used to estimate the confidence interval for the one-sample *t*-values as follows^13,18^, thus removing the requirement for unverifiable assumptions on the probability distribution of the data. The *t*-values of each individual subject were re-computed using two bootstrapped re-samples obtained from *PSI*_*l*,*m*_(*f*, *t*) in the 2-back and 0-back conditions. The one-sample *t*-test was then performed on the bootstrapped two-sample *t*-values to obtain statistical t-value across the subjects. The bootstrap procedure was repeated 2000 times to generate a distribution of the statistical t-values that allowed determination of the threshold of the *t*-value (bootstrap p < 0.05) (Fig. 1). The number of electrode pairs with a *t*-value greater than the threshold was counted for each frequency.

To test the significance of the difference in the number of significant electrode pairs (i.e., electrode pairs with significant modulation of PSIs) between the depression and control subjects for the delta band (1–3 Hz), theta band (4–7 Hz), alpha band (8–13 Hz), and beta band (14–30 Hz), a bootstrap procedure was used. The bootstrapped depression and control subjects were re-sampled from all the subjects in both groups, and the difference in the number of significant electrode pairs between the surrogated groups was re-computed for each frequency band. The bootstrap procedure was repeated 1000 times to generate a distribution for the difference that allowed determination of significance of the difference in the real number of significant electrode pairs.

To test the significance of the difference in the task-dependent modulation of the PSIs of significant electrode pairs between the depression and control groups, the sums and average values of the PSIs increase/decrease of all significant electrode pairs were compared between the depression and control groups for each frequency using two-sample *t*-tests.

### EEG Phase Synchronization Clustering

The significant electrode pairs were categorized using correlation coefficients (Fig. 1). Firstly, the correlation coefficients of the PSI time series between every two electrode pairs were computed for each subject. The overall mean of the correlation coefficients across the subjects was then calculated. Subsequently, difference between the correlation coefficients of every two significant electrode pairs and the overall mean was examined by using one sample *t*-tests across the subjects (one-tailed p < 0.05). On the basis of the correlation coefficients of the PSI time series, the significant electrode pairs were categorized into a set of clusters^14^. In particular, the two significant electrode pairs were categorized into the same cluster if the PSIs between them had a correlation coefficient higher than the overall mean. In other words, in a resultant cluster, each electrode pair had significant correlation of PSI with at least one other electrode pair (i.e., their correlation coefficient was significantly higher than the overall mean), and electrode pairs in one cluster did not have significant correlations of PSI with electrode pairs in any other cluster. In addition to a threshold for the significance of the correlation coefficient, a threshold for the number of electrode pairs within a cluster was used, to reduce the risk of a type I error. A statistical procedure was performed to determine the threshold of the number of electrode pairs within a cluster as follows. (1) 100 electrode pairs was randomly taken from all electrode pairs without replacement; (2) a group of subjects was randomly taken from the depression and control groups; (3) a frequency value *f* was randomly sampled from all frequencies between 0–30 Hz; (4) the sampled 100 electrode pairs were clustered on the basis of correlation coefficients of the *PSI*_*l*,*m*_(*f*, *t*) between them. The number of electrode pairs within the cluster was obtained; (5) the above procedure was repeated 1000 times to generate a distribution of the cluster sizes that allowed determination of the threshold of the numbers of electrode pairs within a cluster (bootstrap p < 0.05).

### Correlation Analysis of PSI

The time series of PSIs at the frequencies of interest in the 2-back task were used to compute the correlation coefficient with the following equation:

4 $$r(\tau )=\frac{\frac{1}{N}\sum _{i=1}^{N}({f}_{i}-\bar{f})\times ({g}_{i+\tau }-\bar{g})}{\sqrt{\frac{1}{N}\sum _{i=1}^{N}{({f}_{i}-\bar{f})}^{2}\times \sum _{i=1}^{N}{({g}_{i}-\bar{g})}^{2}}}$$

where *r*(*τ*) is the correlation coefficient at the time lag *τ* between two PSI time series *f*_*i*_ and *g*_*i*_; *N* is the number of time points in the time series, and $\bar{f}$ and $\bar{g}$ are the mean values of the time series *f*_*i*_ and *g*_*i*_ respectively.

The correlation coefficient *r*(0) between two PSI time series was utilized for PSI clustering. Additionally, to quantify the average correlation coefficient of electrode pairs within a cluster, all correlation coefficients computed over the time series of PSIs in the cluster were averaged on an intra-subject basis. The averaged correlation coefficients were then averaged across the subjects.

### Cluster-based Statistical analysis

For each obtained phase synchronization cluster, the cluster value was derived from the sum of statistical *t*-values of all significant electrode pairs in the cluster. Then, the cluster values were used for a permutation test to examine the significance of effects (Fig. 1). The permutation was executed 1000 times. In each permutation, the *PSI*_*l*,*m*_(*f*, *t*) in the 0-back and 2-back conditions were interchanged. Then, two-sample *t*-tests for each individual subjects followed by one-sample *t*-test across the subjects were re-performed to obtain statistical *t*-values for all electrode pairs in the cluster, and the cluster value was acquired accordingly. For all 1000 permutations, a new distribution of the cluster values was determined. Based on this distribution, the significance of the initial cluster value can be determined. Therefore the p-values resulting from the permutation test were corrected for multiple comparisons. If a cluster cannot survive the permutation test, we will use a stringent threshold (e.g. p < 0.01) to re-determine significant electrode pairs (Fig. 1) followed by the re-clustering and cluster-based statistical procedures again.

Because there might be multiple clusters for significance testing, we used a Holm-Bonferroni method to control the family-wise error rate (p < 0.05).

## Results

### Behavioral data

For the 0-back task, neither response accuracy (*t*_63_ = 1.740, *p* = 0.092) nor reaction time (*t*_63_ = 0.766, *p* = 0.449) showed a significant difference between the two groups. Similarly, response accuracy (*t*_63_ = 1.650, *p* = 0.110) and reaction times (*t*_63_ = 0.536, *p* = 0.596) for the 2-back task did not differ significantly between the two groups.

### Phase synchronization results

**Figure 2:**
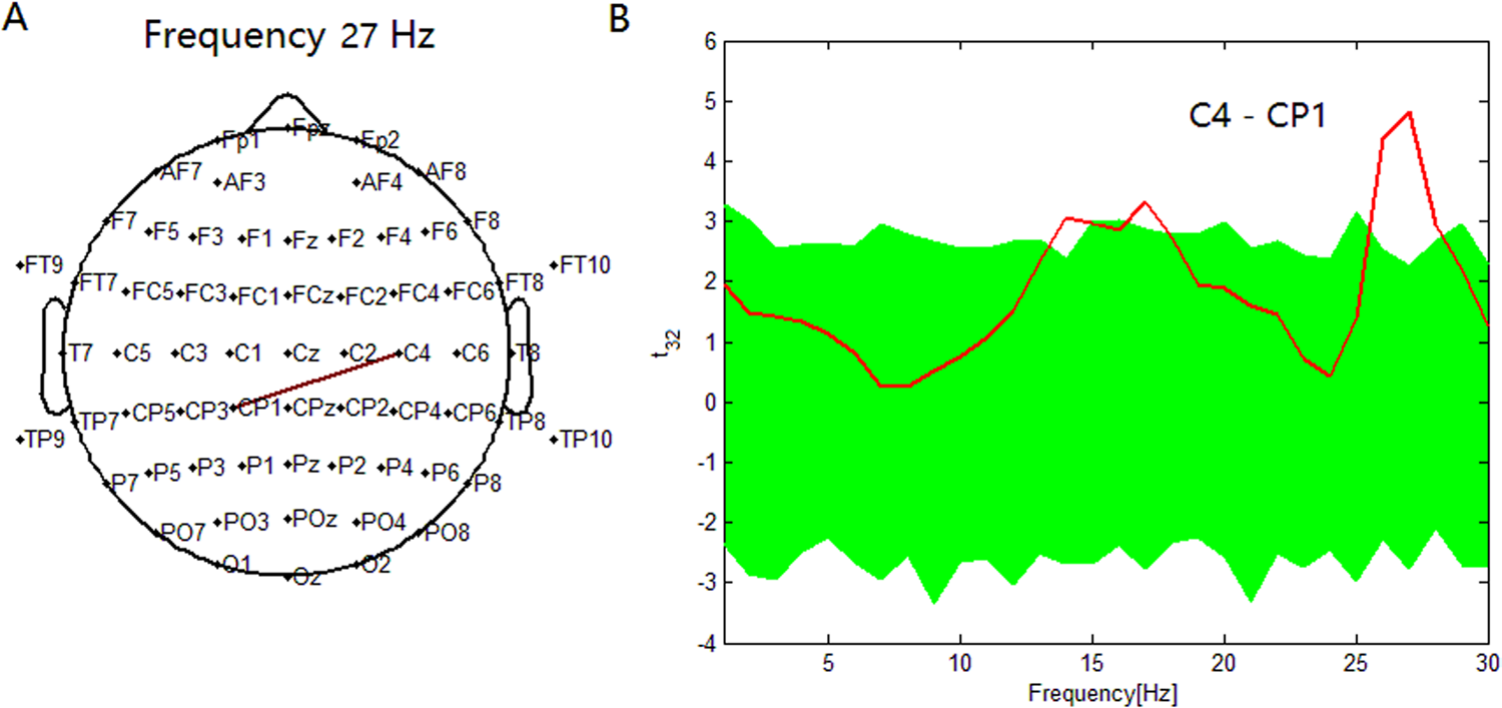
In the depression group. (**A**) Maximum significance of PSI increase during the 2-back condition compared to the 0-back condition was found between C4 electrode and CP1 electrode at 27 Hz. (**B**) t statistical values for the difference of the PSI between the 2-back condition and 0-back condition for the pair (C4-CP1) across subjects. At this pair, the PSI was higher in the 2-back condition than in the 0-back condition with a peak at 27 Hz. Greenband represents the 95% confidence interval constructed using the bootstrap method. Red line represents the t values of one sample t-test.

The pairs of electrodes that showed significant PSI modulations between the 2-back and 0-back conditions from 1 Hz to 30 Hz (P < 0.05) were counted. For example, in the depression group, the maximum significance of PSI increase was found between the C4 and CP1 electrodes at 27 Hz (Fig. 2A,B).

**Figure 3:**
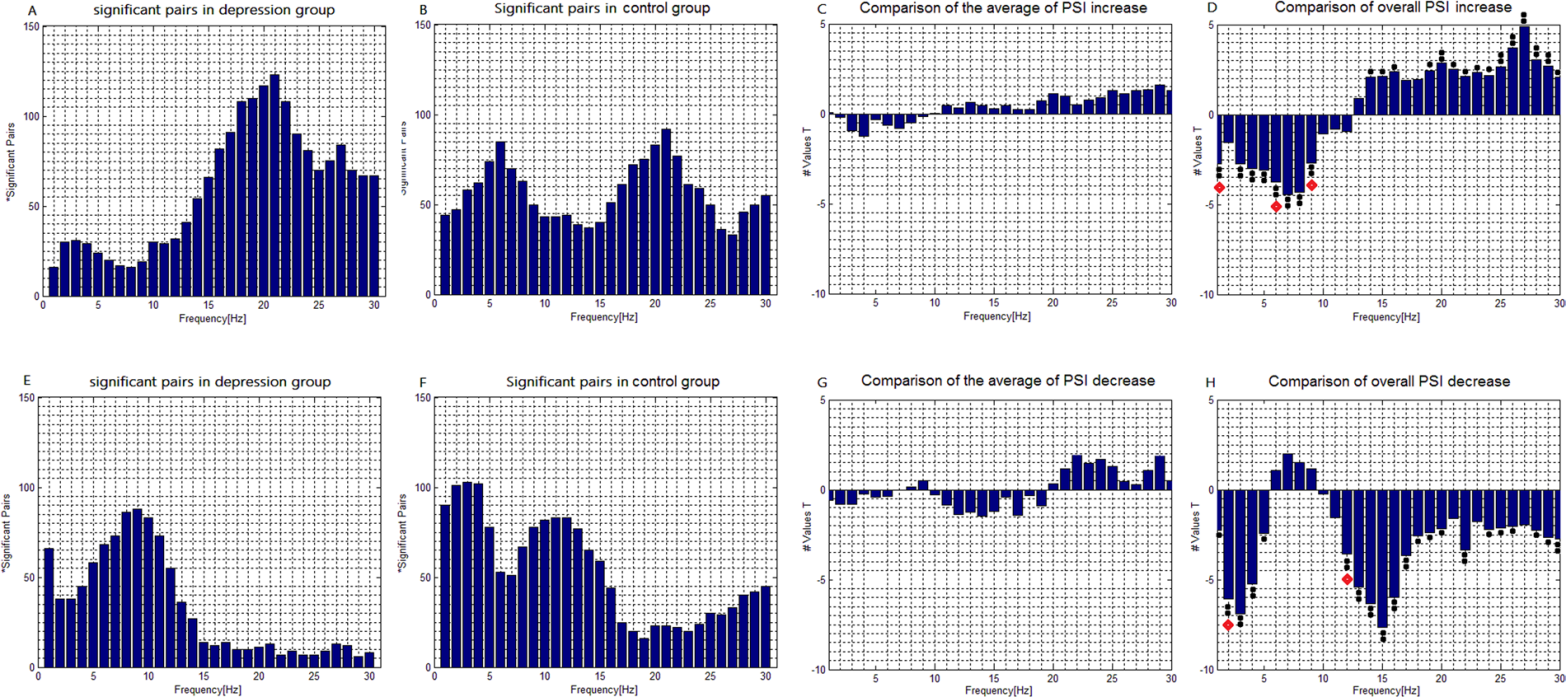
Task-dependent increase and decrease of EEG phase synchronization indices across all subjects. (**A**) The number of electrode pairs exhibiting greater PSI during the 2-back condition than that during the 0-back condition in the depression group. (**B**) The number of electrode pairs exhibiting greater PSI during the 2-back condition than that during the 0-back condition in the control group. (**C**) Comparison of the average value of the PSI increase among all significant pairs in the depression group with that in the control group using a two-sample t-test. (**D**) Comparison of the overall PSI increase among all significant pairs in the depression group with that in the control group using a two-sample t-test. (**E**) The number of electrode pairs exhibiting lower PSI during the 2-back condition than that during the 0-back condition in the depression group. (**F**) The number of electrode pairs exhibiting lower PSI during the 2-back condition than that during the 0-back condition in the control group. (**G**) Comparison of the average value of the decrease of PS among all significant pairs in the depression group with that in the control group using a two-sample t-test. (**H**) Comparison of the overall PSI decrease among all significant pairs in the depression group with that in the control group using a two-sample t-test. Note:  indicates p < 0.05 (uncorrected) and  indicates p < 0.01 (uncorrected). Besides the most significant frequencies in the delta, theta, alpha and beta bands, the frequencies marked with  were also used for clustering.

In the depression group, the number of electrode pairs exhibiting a significant PSI increase in the beta band was larger than that of the control group (*p* < 0.01). Conversely, in the delta, theta, and alpha bands, the number was significantly lower in the depression group than in the control group (all with *p* < 0.01; Fig. 3A,B). The numbers of electrode pairs exhibiting a significant PSI decrease in the delta, theta, and beta bands were lower in the depression group than in the control group (all with *p* < 0.01; Fig. 3E,F).

We also investigated differences in PSI modulation between the two groups for all significant pairs. The sum of PSI increase among all significant pairs in the depression group was higher than that in the control group in the beta (e.g. at 27 Hz, *t*_63_ = 4.8860, *p* < 0.001) band (Fig. 3D), but was lower than that in the control group in the delta (e.g. at 3 Hz, *t*_63_ = 2.7421, *p* < 0.01), theta (e.g. at 7 Hz, *t*_63_ = 4.4536, *p* < 0.001) and alpha (e.g. at 8 Hz, *t*_63_ = 4.3080, *p* < 0.001) bands. There was no significant group effect on the average value of the PSI increase among all significant pairs (Fig. 3C). Additionally, in cases where the subjects showed a significant PSI decrease, the sum of PSI decrease among all significant pairs in the depression group (Fig. 3H) was lower than that in the control group in the delta (e.g. at 3 Hz, *t*_63_ = 6.9211, *p* < 0.001), theta (e.g. at 4 Hz, *t*_63_ = 5.2431, *p* < 0.001), alpha (e.g. at 13 Hz, *t*_63_ = 5.4367, *p* < 0.001) and beta (e.g. at 15 Hz, *t*_63_ = 7.6551, *p* < 0.001) bands. There was no significant group effect on the average value of the PSI decrease (Fig. 3G). The PSIs at the above frequencies in the delta, theta, alpha and beta bands that showed the most significant difference were used for clustering. Additionally, because the frequencies may not be adequately discriminated from each other using the wavelet convolution methods, the PSIs at some adjacent frequencies that showed significant differences (*p* < 0.01) were also selected for clustering.

### Topology of Clusters with PSI Increase

A threshold of 9 electrode pairs within a cluster was obtained by the statistical procedure.

#### Delta Phase Synchronization

**Figure 4:**
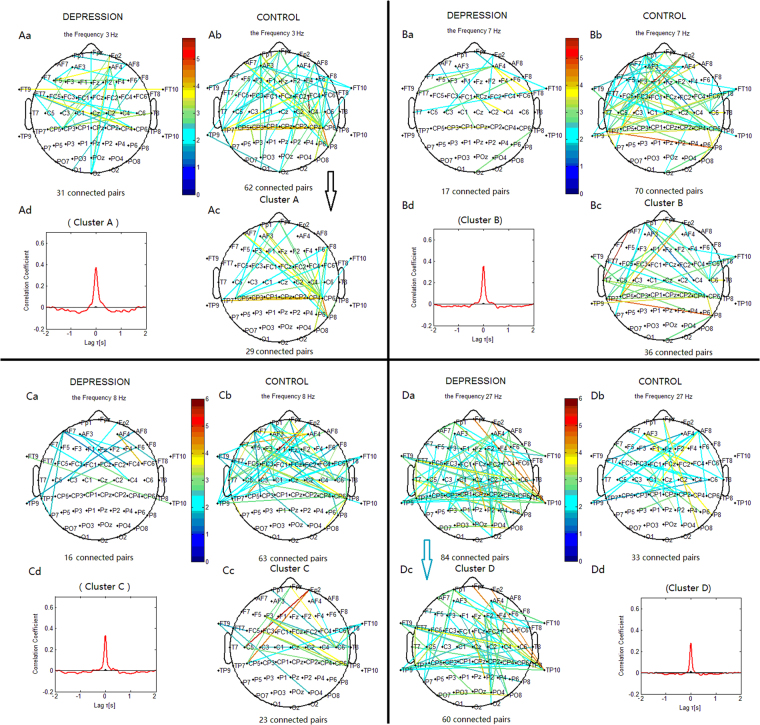
Clustering of the most significantly increased phase synchronization indices in the delta, theta, alpha, and beta bands for both the depression and control groups based on the correlation coefficient of phase synchronization indices during the 2-back task. Lines represent significant PSI increase during the 2-back condition relative to that during the 0-back condition (P < 0.05). (Aa, Ba, Ca, Da) Significant PSI increase for the depression group. (Ab, Bb, Cb, Db) Significant PSI increase for the control groups. Ac: Cluster A. Bc: Cluster B. Cc: Cluster C. Dc: Cluster D. Cluster A, B and C identified in the control group were significant using a control of family-wise error rate at the level of α = 0.05; Cluster D identified in the depression group was significant with *p* < 0.05 (corrected for multiple comparisons). Drawing is the top view of the scalp. Each black spot signifies an electrode that was used for measurement. (Ad, Bd, Cd and Dd) are correlation coefficient of phase synchronization within corresponding clusters.

**Figure 5:**
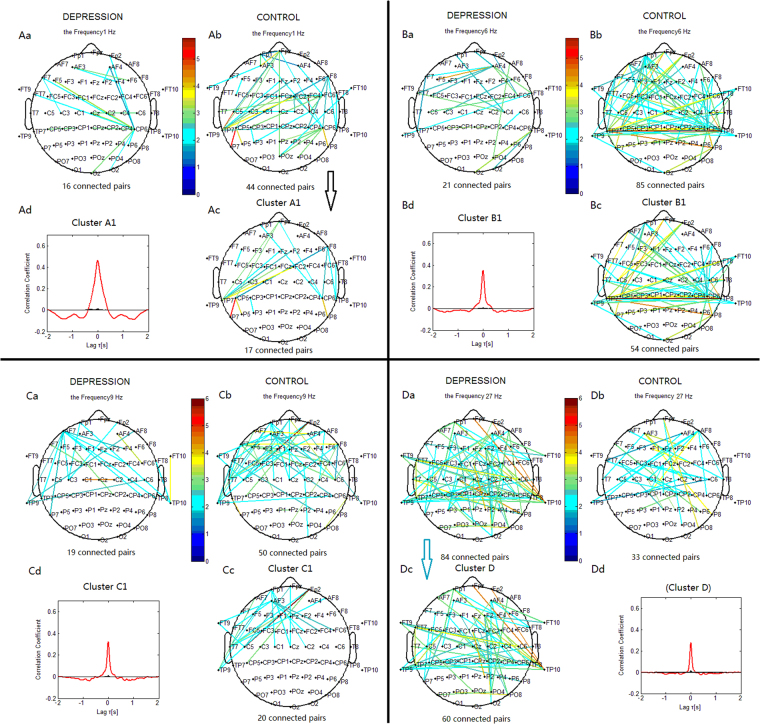
Clustering of some significantly increased phase synchronization indices in delta, theta, alpha, and beta bands for both the depression and control groups. Lines represent significant PSI increase during the 2-back condition relative to that during the 0-back condition (*p* < 0.05). (Aa, Ba, Ca, Da) Significant PSI increase for the depression group. (Ab, Bb, Cb, Db) Significant PSI increase for the control groups. Ac: Cluster A1. Bc: Cluster B1. Cc: Cluster C1. Dc: Cluster D. Cluster A1, B1 and C1 identified in the control group were significant using a control of family-wise error rate at the level of α = 0.05; Cluster D identified in the depression group was significant with *p* < 0.05 (corrected for multiple comparisons).

For phase synchronization at 3 Hz, the connections in the control group could be classified into one significant cluster. This cluster ‘A’ (Fig. 4Ac) linked 29 pairs of electrodes, and primarily included long-range connections between the left frontal and right centroparietal sites, and between the right frontal and left centroparietal sites. It also included interhemispheric centroparietal connections. For phase synchronization at 1 Hz, the cluster ‘A1’ (Fig. 5Ac) included connections between the frontal and bilateral centroparietal or temporoparietal sites. The connections in the depression group (Figs 4Aa and 5Aa) could not be classified into one cluster. The control group showed more connections between the frontal and centroparietal sites, and more interhemispheric centroparietal connections than the depression group.

#### Theta Phase Synchronization

For the phase synchronization at 7 Hz, the connections in the control group could be classified into one significant cluster. This cluster ‘B’ (Fig. 4Bc) linked 36 pairs of electrodes, and primarily included connections between the left frontal sites and right centroparietal sites, and between the frontopolar sites and left centroparietal sites. It also included interhemispheric centroparietal connections. For phase synchronization at 6 Hz, the connections of the cluster ‘B1’ (Fig. 5Bc) were similar to those of the cluster ‘B’. The connections in the depression group (Figs 4Ba and 5Ba) could not be classified into one cluster. The control group showed more connections than the depression group between the frontal and centroparietal scalp sites and more interhemispheric centroparietal connections.

#### Alpha Phase Synchronization

For the phase synchronization at 8 Hz, the connections in the control group could be classified into one significant cluster. This cluster ‘C’ (Fig. 4Cc) linked 23 pairs of electrodes, and primarily included connections between the left frontal and right centroparietal sites, and between the right frontal and left centroparietal sites. For phase synchronization at 9 Hz, the cluster ‘C1’ (Fig. 5Cc) included connections between the frontal and left central or temporoparietal sites, and between the left frontal and right central or frontocentral sites. The connections in the depression group (Figs 4Ca and 5Ca) could not be classified into one cluster. The depression group exhibited less links between the frontal and bilateral centroparietal sites than the control group.

#### Beta Phase Synchronization

For the phase synchronization at 27 Hz, the connections in the depression group could be classified into one significant cluster. This cluster ‘D’ (Fig. 4Dc) linked 60 pairs of electrodes, and primarily included connections between the frontal and right temporoparietal or centroparietal sites, between the frontocentral and left temporoparietal sites, and within the frontal sites. There were also interhemispheric temporoparietal connections. The connections in the control group could not be classified into one cluster. The depression group showed more connections than the control group between the frontal and temporoparietal or centroparietal sites, more connections within the frontal sites, and more interhemispheric temporoparietal connections.

### Topology of Clusters showing a PSI Decrease

#### Delta Phase Desynchronization

**Figure 6:**
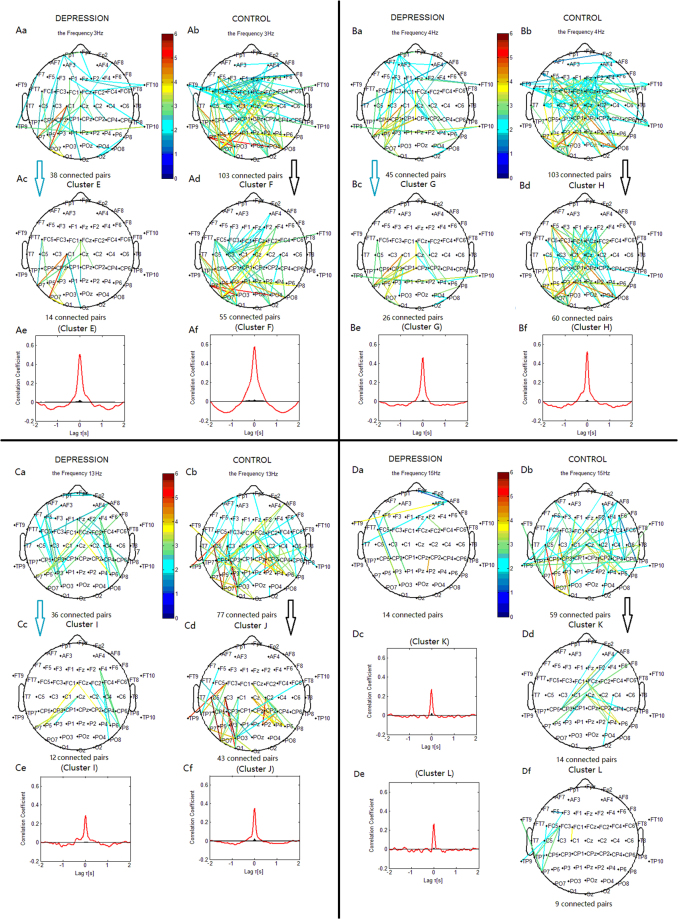
Clustering of the most significantly decreased phase synchronization indices in delta, theta, alpha, and beta bands for both depression and control groups based on the correlation coefficient of phase synchronization indices during the 2-back task. Lines represent significant PSI decrease during the 2-back condition relative to that during the 0-back condition (P < 0.05). (Aa, Ba, Ca, Da) Significant PSI decrease for the depression group. (Ab, Bb, Cb, Db) Significant PSI decrease for the control group. Ac: Cluster E. Ad: Cluster F. Bc: Cluster G. Bd: Cluster H. Cc: Cluster I. Cd: Cluster J. Dd: Cluster K. Df: Cluster L. Cluster E, G and I identified in the depression group were significant using a control of family-wise error rate at the level of α = 0.05; Cluster F, H, J, K and L identified in the control group were significant using a control of family-wise error rate at the level of α = 0.05. Ae, Af, Be, Bf, Ce, Cf, Dc and De are correlation coefficient of phase synchronization within corresponding clusters. Note: The sum of the PSI decreases among all significant electrode pairs was compared between Cluster E and F, between Cluster G and H, and between Cluster I and J using two-sample *t*-tests. The overall PSI decrease among all significant pairs in Cluster E was lower than that in Cluster F (*t*_63_ = 4.4536, *p* < 0.001), in Cluster G was lower than that in Cluster H (*t*_63_ = 4.3080, *p* < 0.001), and in Cluster I was lower than that in Cluster J (*t*_63_ = 4.8860, *p* < 0.001).

**Figure 7:**
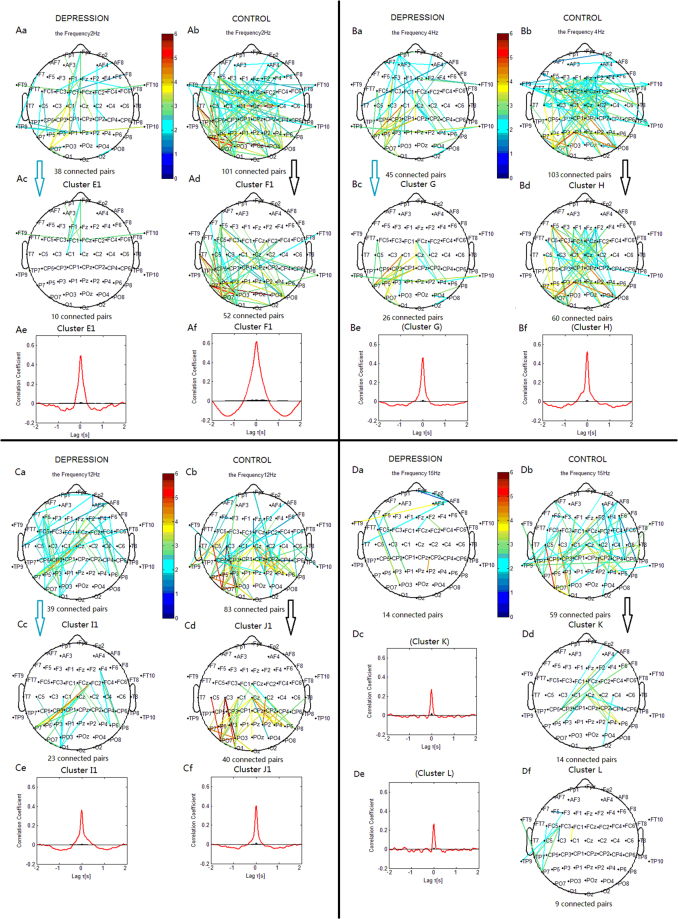
Clustering of some significantly decreased phase synchronization indices in delta, theta, alpha, and beta bands for both the depression and control groups. Lines represent significant PSI decrease during the 2-back condition relative to that during the 0-back condition (*p* < 0.05). (Aa, Ba, Ca, Da) Significant PSI decrease for the depression group. (Ab, Bb, Cb, Db) Significant PSI decrease for the control group. Ac: Cluster E1. Ad: Cluster F1. Bc: Cluster G. Bd: Cluster H. Cc: Cluster I1. Cd: Cluster J1. Dd: Cluster K. Df: Cluster L. Cluster E1, G and I1 identified in the depression group were significant using a control of family-wise error rate at the level of α = 0.05; Cluster F1, H, J1, K and L identified in the control group were significant using a control of family-wise error rate at the level of α = 0.05. Note: The sum of the PSI decreases among all significant electrode pairs was compared between Cluster E1 and F1, between Cluster G and H, and between Cluster I1 and J1 using two-sample *t*-tests. The overall PSI decrease among all significant pairs in Cluster E1 was lower than that in Cluster F1 (*t*_63_ = 5.2530, p < 0.001), in Cluster G was lower than that in Cluster H (*t*_63_ = 4.3080, p < 0.001), and in Cluster I1 was lower than that in Cluster J1 (*t*_63_ = 4.130, p < 0.001).

For the phase desynchronization at 3 Hz, the connections in the depression group could be classified into one significant cluster. This cluster ‘E’ (Fig. 6Ac) linked 14 pairs of electrodes, and primarily included connections between the left central and left parietal or parieto-occipital sites. In the control group, the connections could be classified into cluster ‘F’. This cluster (Fig. 6Ad) linked 55 pairs of electrodes, and primarily included connections between the frontocentral and parieto-occipital or occipital sites, and connections within the left frontocentral region. For the phase desynchronization at 2 Hz, the cluster ‘E1’ (Fig. 7Ac) included connections within the frontal regions. The connections of the cluster ‘F1’ (Fig. 7Ad) were similar to those of the cluster ‘F’.

The control group showed more connections in both hemispheres between the frontocentral sites and parieto-occipital or occipital sites, and more connections within the left frontocentral regions than the depression group.

#### Theta Phase Desynchronization

For the phase desynchronization at 4 Hz, the connections in the depression group could be classified into one significant cluster. This cluster ‘G’ (Fig. 6Bc) linked 26 pairs of electrodes, and primarily included connections between the central or centroparietal and parietal or parietal-occipital sites in the left hemisphere, and between the middle frontocentral or central and right parietal sites. In the control group, the connections could be classified into cluster ‘H’. This cluster (Fig. 6Bd) linked 60 pairs of electrodes, and primarily included connections between the frontal or frontocentral and right parieto-occipital or occipital sites, between the right frontocentral or central and left parieto-occipital sites, and between the middle frontal or frontocentral and centroparietal regions. The depression group showed less links than the control group between the frontal or frontocentral and parieto-occipital or occipital sites, and less links between the middle frontal or frontocentral and centroparietal regions.

#### Alpha Phase Desynchronization

For the phase desynchronization at 13 Hz, the connections in the depression group could be classified into one significant cluster. This cluster ‘I’ (Fig. 6Cc) linked 12 pairs of electrodes, and primarily included connections between the middle central and left parietal sites, and between the right frontocentral and right parietal sites. In the control group, the connections could be classified into cluster ‘J’. This cluster (Fig. 6Cd) linked 43 pairs of electrodes, and primarily included connections between the middle central regions and right parietal sites, and between the left central or centroparietal and left parieto-occipital or parietal sites. For the phase desynchronization at 12 Hz, the connections of the cluster ‘I1’ (Fig. 7Cc) were similar to those of the cluster ‘I’, and the connections of the cluster ‘J1’ (Fig. 7Cd) were similar to those of the cluster ‘J’.

The depression group showed less links than the control group between the left central or centroparietal and left parieto-occipital or parietal sites, and between the middle central and right parietal sites.

#### Beta Phase Desynchronization

For the phase desynchronization at 15 Hz, the connections in the depression group could not be classified into one cluster. In the control group, the connections could be classified into cluster ‘K’ and ‘L’. The cluster ‘K’ (Fig. 6Dd) linked 14 pairs of electrodes, and primarily included connections between the middle central regions and bilateral parietal sites, and between the right frontal sites and left centroparietal sites. The cluster ‘L’ (Fig. 6Df) linked 9 pairs of electrodes, and included connections between the left frontocentral sites and temporoparietal or parietal sites. The control group showed more connections than the depression group between the middle central and bilateral parietal sites, and between the right frontal and left centroparietal sites.

## Discussion

In the visual n-back tasks, in comparison with healthy subjects, the depressed patients demonstrated lower enhancement of lower-frequency delta, theta, and alpha phase synchronization, but higher enhancement of beta phase synchronization (Fig. 3D). It is suggested that these differences are related to memory retention and working memory central executive processing. Meanwhile, lower phase desynchronization in the delta, theta, alpha, and beta bands was found in depressed patients (Fig. 3H). We suggest that this reflects impaired ability in cortical inhibition, with abnormally increased beta phase synchronization in major depression being associated with a new functional circuit to repair the impaired abilities in attention control, memory retention, and central executive processing that are required in demanding working memory tasks.

### Phase Synchronization in Control Subjects

Healthy subjects showed a significant PSI increase in the 2-back condition versus the 0-back condition in all oscillation bands (1–30 Hz). This finding was most evident in theta (e.g. 6 Hz) and beta (e.g. 21 Hz) oscillations (Fig. 3), and is consistent with previous studies that reported increased theta phase synchronization as a function of memory demand^14,19,20^, as well as previous reports that increased beta oscillations may be related to attention and top-down control^14,21,22^.

Compared to the 0-back condition, healthy subjects performing the 2-back condition showed enhanced phase synchronization in all frequency ranges, primarily between the prefrontal and bilateral centroparietal sites, and between the left and right centroparietal sites (e.g. cluster A, B, and C; Fig. 4Ac,Bc,Cc), regions that are denoted as the ‘frontoparietal network’. The present findings on this synchronization network are in line with previous studies^3,4^. Additionally, because functional imaging and patient data indicate that central executive functions are associated with frontal areas, whereas short-term memory storage is related to posterior frontal and parietal areas^11,23^, the frontoparietal network may present the framework for short-term memory storage and working memory central executive processing.

### Phase Synchronization in Major Depression

**Figure 8:**
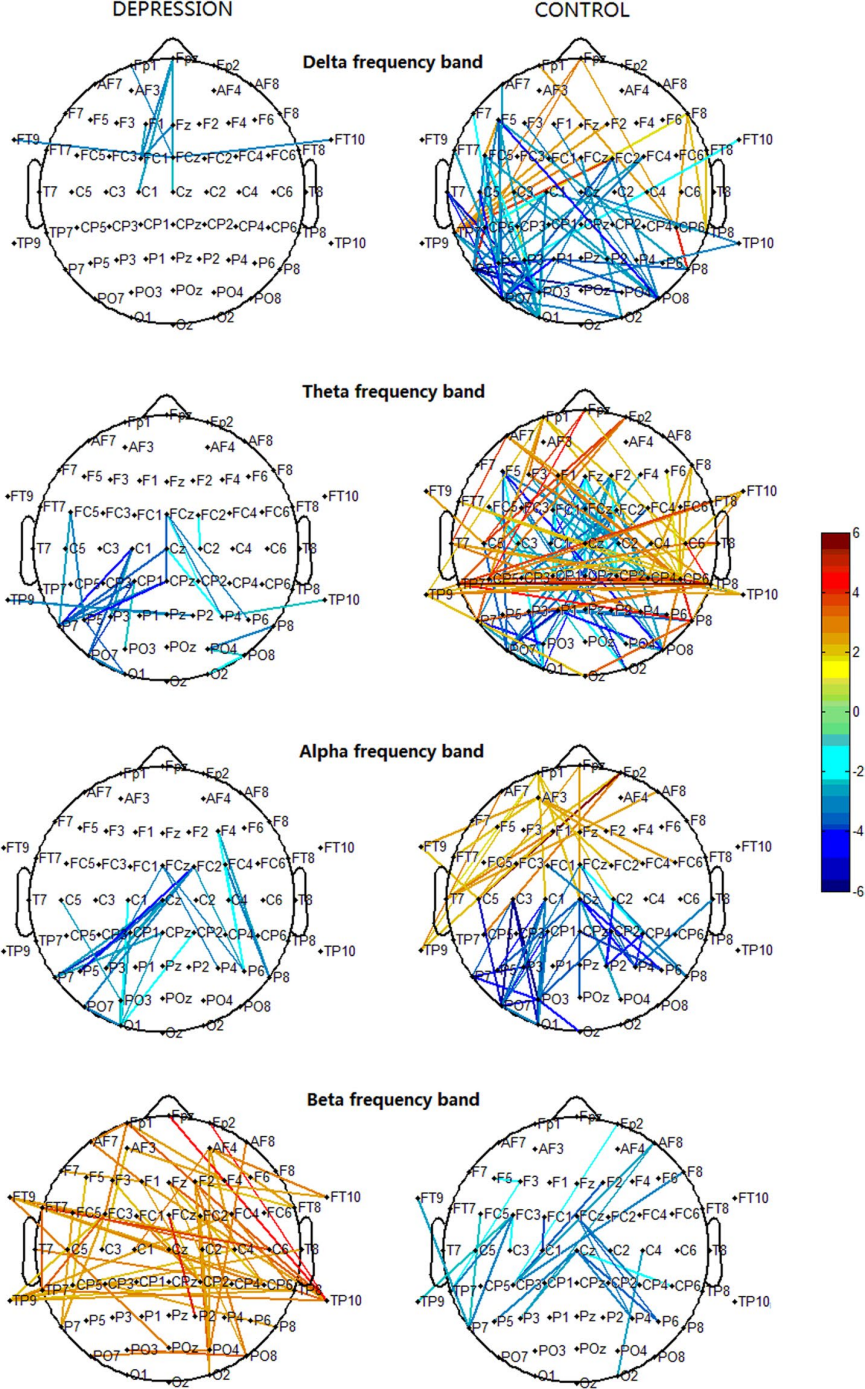
Clusters in the delta, theta, alpha, and beta bands obtained from Figs 5 and 7 showed the significant phase synchronization and desynchronization during the 2-back tasks and the difference between the depression and control subjects. Red lines represent significant phase synchronization; blue lines represent significant phase desynchronization.

In comparison with healthy subjects, depressed patients showed less pronounced enhancement of synchronization in delta, theta, and alpha oscillations (Fig. 3D; Fig. 8), which indicates an impaired ability to implement working memory processing through the frontoparietal circuits by means of these oscillatory activities. In particular, the patients showed the least pronounced enhancement in theta synchronization, while coherent theta oscillations are believed to reflect central executive functions^24–27^ that are important for the interactions between posterior association cortices (where sensory information is thought to be stored) and prefrontal regions (where relevant current information is held and continuously updated). This might reflect impairments in memory retention and working memory central executive processing, which are supposed to result in deteriorations in task performance.

Large alpha oscillations may relate to the inhibition and disengagement of task-irrelevant cortical areas. However, alpha oscillations are also enhanced during the short-term and working memory retention period; short-term and working memory related alpha oscillations in the frontoparietal network during the memory retention period are an essential constituent of the network activity that sustains the neuronal representations of memorized items^28–30^. Accordingly, the present finding of less pronounced alpha synchronization in the frontoparietal network may reflect impaired working memory and short-term memory retention in major depression.

A question can be asked as to how can depressed patients with impaired memory retention and central executive processing functions maintain normal levels of performance in effortful working memory tasks? Compared with healthy subjects, we found that depressed patients showed more pronounced enhancement of beta phase synchronization between the frontal and bilateral temporoparietal sites, and between the left and right temporoparietal sites. This is a similar frontoparietal framework to that found in healthy subjects, but contains more short-range frontal connections. This result can be interpreted within the context of previous studies regarding the role of rhythmic oscillations in regulating brain activity. Lower frequency oscillations operate at a broader level across the brain, binding more distant areas into functional units, while faster (higher frequency, e.g. beta band) oscillations operate at a shorter level, binding local functional units^31^.

Previous studies have reported that beta oscillations may be related to attentional processes (top-down control) and the maintenance of the current cognitive state^21,22,32,33^. In this light, the enhanced beta synchronization in the frontoparietal network may reflect increased attentional control and task engagement in major depression^22^. Furthermore, as we found no significant between-group difference in either performance or reaction time, we suggest that the beyond-normal enhancement of beta phase synchronization plays an important role in compensating for impaired ability in memory retention and working memory central executive processing. This faster synchronous oscillatory network, which includes more interhemispheric temporoparietal connections and short-range frontal connections, presents a new cortical circuit that substitute for disrupted long-range and slower synchronous oscillatory networks in major depression.

### Phase Desynchronization in Control Subjects

For all oscillation frequencies, electrode pairs were also demonstrated a significant PSI decrease in the 2-back condition versus the 0-back condition, which was most evident in delta (e.g. 3 Hz) and alpha (e.g. 11 Hz) oscillations (Fig. 3). Alpha oscillations have shown desynchronization in many tasks^11^, reflecting an increase in attention demands. The Delta frequency band is predominant during deep sleep, and this frequency range is associated with motivational processes and brain reward systems^34,35^. The PSI decrease in delta oscillations may reflect a brain state that was more aroused in the 2-back condition than in the 0-back condition.

Compared with the 0-back condition, healthy subjects in the 2-back condition showed decreased PSI between the central and parieto-occipital sites in all bands (clusters F, H, J, K; Fig. 6). Thus, the attenuated phase synchronization in the centro-parieto-occipital regions was associated with demanding working memory tasks. Both phase synchronization and desynchronization can be interpreted within the context of previous studies which found both blood oxygen level dependent (BOLD) increases in the inferior/middle frontal gyri and inferior/superior parietal lobules, and BOLD decreases in regions including the medial frontal gyrus, precentral gyrus, posterior cingulate gyrus, cuneus, and middle occipital gyrus, as working memory demands increased^4^. Decreased BOLD responses reflecting the suppression of neuronal activities relative to the reference state^13^ are in line with the present findings of decreased phase synchronization. We propose that the demanding working memory processing was physiologically implemented by releasing task-relevant areas from inhibition through phase synchronization, and suppressing task-irrelevant regions through phase desynchronization^30,36^. Therefore, the finding of phase desynchronization may reflect an adjustment through interaction/competition between the centro-parieto-occipital regions and the frontoparietal short-term memory and working-memory related regions, which indirectly contributes to the enhancement of phase synchronization in the short-term memory and working-memory related frontoparietal network^37,38^.

Additionally, in a previous n-back memory study, alpha and beta ERD were observed in posterior recording sites, and these rose in association with increasing working memory load^39^. Consistently, our finding of alpha and beta phase desynchronization in the centro-parieto-occipital regions demonstrated increased attention/memory demands, while delta phase desynchronization may reflect a more aroused brain state.

### Phase Desynchronization in Major Depression

Compared with healthy subjects, depressed patients showed less pronounced attenuation of phase synchronization in all bands in the central-parieto-occipital regions (Fig. 3H; Fig. 8). Less pronounced delta and alpha phase desynchronization might be related to inadequate cortical inhibition. Based on our aforementioned interpretation, less pronounced phase desynchronization in depression may lead to reduced enhancement of phase synchronization in delta, theta, and alpha oscillations, due to insufficient suppression of the centro-parieto-occipital regions and release of the frontoparietal network from inhibition. While the enhancement of phase synchronization was insufficient, particularly in theta and alpha oscillations, the beyond-normal enhancement of beta phase synchronization was able to create a new functional pathway for memory retention and central executive processing.

In summary, the insufficient phase desynchronization in the centro-parieto-occipital regions may indicate that depressed individuals suffered impaired cortical inhibition abilities, and that the reduced inhibitory control impacts negatively upon working memory and attention efficiency^1,2,40^. New functional connections by means of faster beta oscillations and their synchronous activities were created to maintain indispensable cognitive control. However, this functional compensation by employing more short-range frontal and interhemispheric temporoparietal connections may impair cognitive capacity in the brain, which implies a deterioration of flexibility in cognitive processing.

## Conclusion

In the visual n-back task, although the insufficient phase synchronization and desynchronization in major depression were identified reflecting impaired memory and attention efficiency, abnormally enhanced beta phase synchronization containing more short-range frontal connections and interhemispheric temporoparietal connections, reflects the creation of new cortical circuits to repair the memory retention and working-memory central executive functions. This adaptive compensatory mechanism is also reflected in a deterioration of flexibility in cognitive control.
